# Depression among Armed Police Force Soldiers Serving in a Police Headquarter: A Descriptive Cross-sectional Study

**DOI:** 10.31729/jnma.7497

**Published:** 2022-05-31

**Authors:** Nidesh Sapkota, Atit Tiwari, Mandeep Kunwar, Nisha Manandhar, Bharat Khatri

**Affiliations:** 1 Department of Psychiatry, Patan Academy of Health Sciences, Lagankhel, Lalitpur, Nepal; 2 Department of Psychiatry, BP Koirala Institute of Health Sciences, Dharan, Sunsari, Nepal; 3 Department of Community Medicine and Tropical Diseases, BP Koirala Institute of Health Sciences, Dharan, Sunsari, Nepal; 4 Sukraraj Tropical and Infectious Disease Hospital, Teku, Kathmandu, Nepal

**Keywords:** *armed forces personnel*, *depression*, *depressive symptoms*, *soldiers*

## Abstract

**Introduction:**

Depression is a common mental health problem among soldiers worldwide. Depression decreases the efficiency and productivity of the soldiers. The objective of this research is to find out the prevalence of depression among armed police force soldiers serving in a police headquarter.

**Methods:**

A descriptive cross-sectional study was done on a total of 314 soldiers serving in the eastern regional armed police force headquarters of Nepal from January 15, 2017 to June 14, 2017 after receiving ethical clearance was taken from the Institutional Review Committee (Reference number: 140/073/074-IRC). Convenience sampling was done. Beck Depression Inventory was used to assess the prevalence of depression. Data were collected and entered in the Statistical Package for the Social Science version 15.0. which was used for data analysis. Point estimate at 95% Confidence Interval was calculated along with frequency and percentage for binary data.

**Results:**

Among 314 soldiers, 133 (42.36%) (36.89-47.83 at a 95% Confidence Interval) soldiers had depression. Out of 132, 47 (14.97%) had mild mood disturbance, 33 (10.51%) had borderline depression, 40 (12.74%) had moderate depression, 10 (3.18%) had severe depression and 3 (0.96%) had extreme depression.

**Conclusions:**

The prevalence of depression in our study was higher when compared to other studies conducted in similar settings.

## INTRODUCTION

A soldier's life is exposed to various physical and mental stresses and thus becomes vulnerable to different mental health problems. Depression has been identified as a common mental health problem in the military worldwide.^1,2^ Depression decreases the efficiency and productivity of the soldiers.

Nepalese Armed Police Force soldiers not only contribute to the internal and border security of the nation but also actively serve in United Nations peacekeeping missions.^3^ The nation needs to know about the mental health issues of its soldiers so that proper policies and interventions will be carried out to address the issues. However, there is a paucity of data regarding the prevalence of depression among Nepalese soldiers.

The objective of this research is to find out the prevalence of depression among armed police force soldiers serving in a police headquarter of eastern region.

## METHODS

This was a descriptive cross-sectional study conducted in Eastern Regional Armed Police Force Headquarters, Baraha Brigade in Pakali, Sunsari, Nepal. After obtaining the ethical clearance from Institutional

Review Committee (Reference number: 140/073/074-IRC), the study was commenced and conducted from January 15, 2017 to June 14, 2017. Meanwhile, the permission to study in the field was also taken from the Eastern Regional Armed Police Force headquarters and Eastern Regional Armed Police Force hospital. The soldiers present at headquarter who had already completed their recruit training and gave informed written consent to participate in the study were included whereas the soldiers who were uncooperative or did not consent were excluded from the study. Convenience sampling was done. The sample size was calculated using the formula,

n = (Z^2^ × p × q) / e^2^

  = (1.96^2^ × 0.25 × 0.75) / 0.05^2^

  = 289

Where,

n = minimum required sample size,
z = 1.96 at 95% Confidence Interval (CI),
p = prevalence of depression among the soldiers, 25.2%^4^
q = 1-p
e = margin of error, 5%

The required sample size was 289. However, 314 soldiers were enrolled in the study. After explaining the purpose of the study and the confidentiality of data collection, informed consent was obtained from each participant. The proforma containing sociodemographic characteristics of the participant and the Beck Depression Inventory^5^ were given to the participants to be filled. Those soldiers who were assessed as having severe to extreme depressive symptoms were counselled and helped to seek proper mental health services.

Statistical Package for the Social Sciences (SPSS) version 15.0 was used for data entry and analysis. Point estimate at 95% Confidence Interval was calculated along with frequency and percentage for binary data.

## RESULTS

Out of 314 soldiers, 133 (42.36%) (36.89-47.83 at 95% Confidence Interval) soldiers had depression where, 47 (14.97%) had mild mood disturbance, 33 (10.51%) had borderline depression, 40 (12.74%) had moderate depression, 10 (3.18%) had severe depression and 3 (0.96%) had extreme depression (Table 1).

**Table 1:** Categories of depression among the soldiers (n = 133).

Categories	n (%)
Mild mood disturbance	47 (14.97)
Borderline depression	33 (10.51)
Moderate depression	40 (12.74)
Severe depression	10 (3.18)
Extreme depression	3 (0.96)

Among 133 soldiers who had depressive symptoms, 123 (92.48%) were males and 10 (7.51%) were females. Majority of males 43 (32.33%) and females 4 (3%) soldiers had only mild mood disturbance. Extreme depression was found only in 3 (2.25%) of male soldiers. Similarly, most of the soldiers 44 (33.08%) having depressive symptoms, were between 3034 years of age. One hundred six (79.69%) of the soldiers with depressive symptoms were married and the majority 117 (87.96%) followed Hinduism. Seventy-one (53.34%) of the soldiers had studied up to secondary level and the majority 60 (59%) of the soldiers with depressive symptoms had served for greater than 12 years. Similarly, 54 (40.60%) of soldiers with depressive symptoms reported having history of tobacco consumption while 61 (45.86%) of them reported having history of alcohol consumption in their lives (Table 2).

**Table 2:** Socio-demographic characteristics and classification of depression (n = 133).

Characteristics	Categories	Depression n (%)
			Mild	Borderline	Moderate	Severe	Extreme
Age groups	20 - 24	9 (6.76)	6(4.51)	5 (3.75)	2 (1.50)	1 (0.75)
	25 - 29	12 (9.02)	10 (7.51)	11 (8.27)	3 (2.25)	-
(in years)	30 - 34	15 (11.27)	14 (10.52)	12 (9.02)	2 (1.50)	1 (0.75)
	≥35	11 (8.27)	3 (2.25)	12 (9.02)	3 (2.25)	1 (0.75)
Gender	Male	43 (32.33)	31 (23.33)	37 (27.81)	9 (6.76)	3 (2.25)
	Female	4 (3.00)	2 (1.50)	3 (2.25)	1 (0.75)	-
Marital Status	Married	37 (27.81)	26 (19.54)	32 (24.06)	9 (6.76)	2 (1.50)
	Unmarried	10 (7.51)	7 (5.26)	8 (6.01)	1 (0.75)	1 (0.75)
Religion	Hindu	43 (32.33)	29 (21.80)	34 (25.56)	8 (6.01)	3 (2.25)
	Others	4 (3.00)	4 (10.53)	6 (4.51)	2 (1.50)	-
	Primary	6(4.51)	5 (3.75)	8 (6.01)	-	-
Education	Secondary/SLC	28 (21.05)	14 (10.52)	18 (13.53)	9 (6.76)	2 (1.50)
	Above SLC	13 (9.77)	14 (10.52)	14 (10.52)	1 (0.75)	1 (0.75)
Socio-economic	Lower	4 (3.00)	6(4.51)	-	2 (1.50)	_-_
status	Middle	43 (32.33)	27 (20.30)	40 (30.07)	8 (6.01)	3 (2.25)
Service Duration (in years)	<3	9 (6.76)	4 (3.00)	6 (4.51)	1 (0.75)	1 (0.75)
	3-12	20 (15.03)	16 (12.03)	12 (9.02)	3 (2.25)	1 (0.75)
	>12	18 (13.53)	13 (9.77)	22 (16.54)	6 (4.51)	1 (0.75)
Tobacco Intake	Yes	18 (13.53)	15 (11.27)	16 (12.03)	4 (3.00)	1 (0.75)
	No	29 (21.80)	18 (13.53)	24 (18.04)	6 (4.51)	2 (1.50)
Alcohol Intake	Yes	20 (15.03)	15 (11.27)	21 (15.78)	4 (3.00)	1 (0.75)
	No	27 (20.30)	18 (13.53)	19 (14.28)	6 (4.51)	2 (1.50)

## DISCUSSION

Depression is a common mental health problem among the soldiers worldwide.^2,6,9^ Stressors include separation from family, strict and hierarchic disciplinary requirements, deployment in active conflicts, less predictable life, etc. Depression may lead to incomplete training, decrease efficiency, productivity and an overall decrease in quality of life.^10,12^ There is very little knowledge regarding the prevalence of depression among Nepalese soldiers.

Our study on 314 soldiers, showed the prevalence of depression to be 42.36% which was lower when compared to a similar study conducted in Pakistan.^13^ This might be because the latter was conducted mainly among the training recruits where the stress is generally high. However, the prevalence of depression in our study was found to be higher than in some other studies conducted among soldiers.^14,16^ This might be because these studies used different screening tools in assessing the prevalence of depression.

In our study, male soldiers had a higher risk of depression which is in contrast to findings from other similar studies.^15,17^ This might be because the female soldiers in our study were more convenient in sharing their problems with their female colleagues. Also, the depression rate was greater in the higher age group of soldiers. This might be due to an increase in personal and familial responsibilities along with the increase in age. Similarly, soldiers having greater years of active service showed a higher prevalence of depression which is inconsistent with other similar studies.^18^ Our study also showed that about 35.35% of soldiers consumed tobacco products and 35.66% had a history of alcohol consumption in their lives. Different studies conducted elsewhere have also identified tobacco and alcohol consumption to be prevalent among soldiers.^19,22^ It may be because smoking and alcohol consumption are often regarded as the means of relieving stress.^23^

Despite being a major mental health problem among soldiers, very few soldiers reported seeking mental health services for their depressive symptoms, it may be due to the stigma associated with seeking mental health assistance. Similar studies have done elsewhere also reported social stigma as a cause of abstaining from mental health assistance among the soldiers.^24^

The results of the study cannot be generalised as the population under study is limited to soldiers of a single regional headquarter only. Also, because of the descriptive nature of this study, an association between exposure and outcome cannot be made in this study design and risk factors cannot be made out.

## CONCLUSIONS

The prevalence of depression in our study was higher when compared to other studies conducted in similar settings. Nepalese soldiers have been contributing to world peace through the United Nations peacekeeping force, so peace within themselves could raise their efficiency and leave an impact on the world. It is high time, for us to conduct similar research across the nation, to identify the factors associated with depression among soldiers and intervene at both the policy'making level and on a clinical basis in addressing the issues.
